# Up-to-Date Imaging for Parathyroid Tumor Localization in MEN1 Patients with Primary Hyperparathyroidism: When and Which Ones (A Narrative Pictorial Review)

**DOI:** 10.3390/diagnostics15010011

**Published:** 2024-12-25

**Authors:** Valentina Berti, Francesco Mungai, Paolo Lucibello, Maria Luisa Brandi, Carlo Biagini, Alessio Imperiale

**Affiliations:** 1 Nuclear Medicine Unit, Department of Experimental and Clinical Biomedical Sciences, University of Florence, 50134 Florence, Italy; 2 Radiology Unit, Careggi University Hospital, 50134 Florence, Italy; mungaif@aou-careggi.toscana.it; 3 Donatello Bone Clinic, Sesto Fiorentino, 50134 Florence, Italy; plucibello@yahoo.it (P.L.); carlobiagini52@gmail.com (C.B.); 4 Department of Endocrinology, University Vita-Salute San Raffaele, 20132 Milan, Italy; marialuisa@marialuisabrandi.it; 5 Department of Nuclear Medicine and Molecular Imaging, Institut de Cancérologie de Strasbourg Europe (ICANS), University Hospitals of Strasbourg, University of Strasbourg, 67200 Strasbourg, France

**Keywords:** MEN1, hyperparathyroidism, parathyroid tumors, parathyroid imaging, ultrasound, computed tomography, magnetic resonance imaging, fluorocholine, PET

## Abstract

Patients diagnosed with multiple endocrine neoplasia type-1 (MEN1) often initially present with primary hyperparathyroidism (pHPT), and typically undergo surgical intervention. While laboratory tests are fundamental for diagnosis, imaging is crucial for localizing pathological parathyroids to aid in precise surgical planning. In this pictorial review, we will begin by comprehensively examining key imaging techniques and their established protocols, evaluating their effectiveness in detecting abnormal parathyroid glands. This analysis will emphasize both the advantages and potential limitations within the clinical context of MEN1 patients. Additionally, we will explore integrated imaging approaches that combine multiple modalities to enhance localization accuracy and optimize surgical planning—an essential component of holistic management in MEN1 cases. Various imaging techniques are employed for presurgical localization, including ultrasound (US), multiphase parathyroid computed tomography (CT) scanning (4D CT), magnetic resonance imaging (MRI), and nuclear medicine techniques like single photon emission computed tomography/CT (SPECT/CT) and positron emission tomography/CT (PET/CT). US is non-invasive, readily available, and provides high spatial resolution. However, it is operator-dependent and may have limitations in certain cases, such as intrathyroidal locations, the presence of bulky goiters, thyroid nodules, and previous thyroidectomy. Four-dimensional CT offers dynamic imaging, aiding in the identification of enlarged parathyroid glands, particularly in cases of ectopic or supernumerary glands. Despite concerns about radiation exposure, efforts are underway to optimize protocols and reduce doses, including the use of dual-energy CT. MR imaging offers excellent soft tissue contrast without radiation exposure, potentially providing superior differentiation between parathyroid glands and the surrounding structures. Radionuclide imaging, especially PET/CT using radiopharmaceuticals like [^18^F]FCH, shows promising results in localizing parathyroid tumors, particularly in patients with MEN1. [^18^F]FCH PET/CT demonstrates high sensitivity and may provide additional information compared to other imaging modalities, especially in cases of recurrent HPT.

## 1. Introduction

Patients diagnosed with multiple endocrine neoplasia type-1 (MEN1) often present with primary hyperparathyroidism (pHPT) as their initial and most prevalent endocrine disorder. It is not uncommon for patients with MEN1 to have multiple parathyroid adenomas or hyperplasia. This multifocality can pose challenges for diagnosis and treatment as it may require thorough imaging studies and surgical approaches tailored to address all affected glands. Nowadays, the surgery of pHPT in patients with MEN1 is oriented to subtotal parathyroidectomy or less than subtotal parathyroidectomy for the decreased risk of postsurgical hypoparathyroidism [1,2]. For this reason, the localization of pathological parathyroid tissue should be performed with advanced technologies. Notably, individuals with MEN1 can also develop tumors of the thymus; thus, regardless of the chosen surgical method, transcervical thymectomy could be a necessary adjunct [3].

While laboratory tests form the basis of diagnosis, imaging plays a crucial role in localizing parathyroid tumors for precise surgical planning. Normal parathyroid glands are minute, measuring approximately 6 mm in length and 3.5 × 1.5 mm in width, with a flat morphology. They are typically located within the visceral space of the neck, posteriorly or immediately distally to the thyroid lobes and much more rarely within thyroid lobes or ectopically in the mediastinum. Superior parathyroid glands maintain a relatively consistent position, often posterior to the middle third of the thyroid lobes; those with an increased dimension tend anyway to reach the lower pole of the lobes deeper than the thyroid. In contrast, the location of inferior parathyroid glands is more variable, with the majority located caudal and posterior or lateral to the inferior pole of the thyroid lobes. However, they may also be intrathyroidal or within the cervical thymus. They are usually located in a superficial plane right behind the superficial layer of the cervical fascia [4].

Parathyroid tumors may be both small and in ectopic locations, making their detection challenging. Imaging tools rely on identifying hyperfunction or abnormalities in morphology, size, and vascularization. Ultrasound (US) serves as a rapid, reliable, and non-invasive method for identifying abnormally enlarged parathyroid glands in the neck [5,6]. Multiphase parathyroid CT scanning, known as 4D-CT (four-dimensional computed tomography), can rapidly offer precise multiplanar and dynamic imaging, aiding in the accurate localization of enlarged parathyroid glands, particularly when dealing with ectopic or supernumerary glands [7,8]. Magnetic resonance imaging (MRI) offers excellent soft tissue contrast without radiation exposure, providing more precise parathyroid tissue characterization through the acquisition of various imaging parameters that reflect different tissue characteristics [9,10]. Nuclear medicine techniques, such as single photon emission computed tomography/CT (SPECT/CT) and positron emission tomography/CT (PET/CT), combined with various radiopharmaceuticals, offer panoramic views and are essential for detecting hyperfunctioning parathyroid tumors, whether in typical or ectopic positions [11,12].

No single imaging method guarantees the detection of all pathological glands. Therefore, a comprehensive approach, combining multiple modalities, is mandatory for accurate presurgical evaluation in patients with MEN1 with pHPT.

The aim of this pictorial narrative review is to give a comprehensive examination of the imaging techniques used to detect abnormal parathyroid glands in patients with MEN1, including Neck US, CT, MRI, and nuclear medicine imaging. We will examine their diagnostic accuracy, protocols, benefits, and constraints in the clinical context of patients with MEN1, with the scope of suggesting the best integrated imaging approach to enhance localization and surgical intervention strategies.

## 2. Neck Ultrasound

US imaging is a harmless, non-invasive, cost-effective, and powerful tool; however, it suffers from significant variability in accuracy mainly related to operator experience [5,6,13]. Attaining proficient standards mandates operators to optimize their examinations. Operators must possess comprehensive knowledge of neck anatomy and parathyroid embryology. Even medical professionals skilled in US imaging typically need extensive daily practice in the sonography room within a thyroid–parathyroid disease unit, working closely with surgeons and endocrinologists to attain expertise.

Gray-scale sonography requires the use of multi-frequency linear transducers (3–15 MHz) equipped with ecococolor-Doppler and power-Doppler module (Table 1). The procedure entails positioning the patient supine with a hyperextended neck, scanning the anterior and lateral regions of the neck on longitudinal and transversal planes from the mastoid process to the retroclavicular–jugular region.

**Figure 1:**
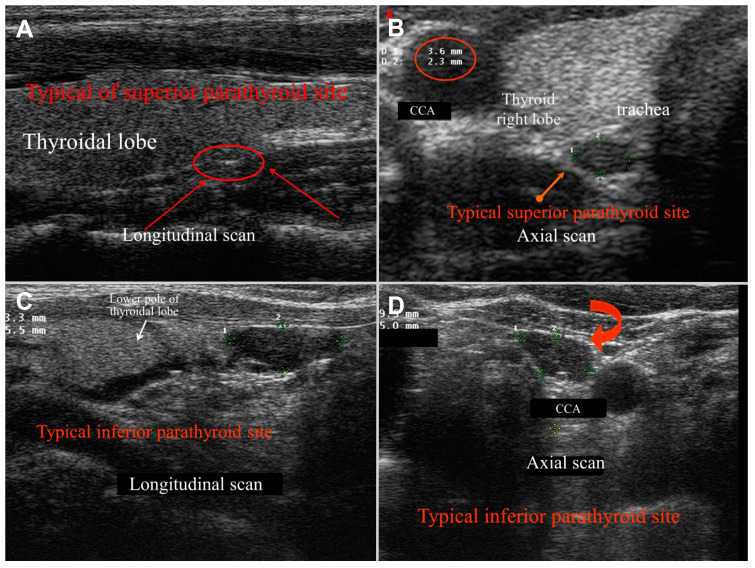
Typical US features and location of superior ((**A**): normal; (**B**): adenoma) and inferior ((**C**): normal; (**D**): adenoma) parathyroids. Superior glands are usually located posteriorly to the third medium of the thyroid lobes, while inferior ones are normally positioned just inferiorly to the lobe poles. The common carotid artery (CCA) represents an important landmark when exploring the thyroid compartment, and inferior parathyroid glands can be often seen along the CCA sheath (as in (**D**)). Parathyroid glands usually present hypoechoic structure and oval shape; one or more glands can be defined as enlarged when the longest diameter measures more than 0.6 cm.

During US examinations, abnormal (pathological) parathyroid glands appear as well vascularized parenchymatous solid masses, very rarely exceeding 3 cm in size. They typically appear hypoechoic compared to the thyroid, exhibiting sharp, regular edges, occasionally with curvilinear features, and seldom containing fluid (Figure 1).

**Figure 2:**
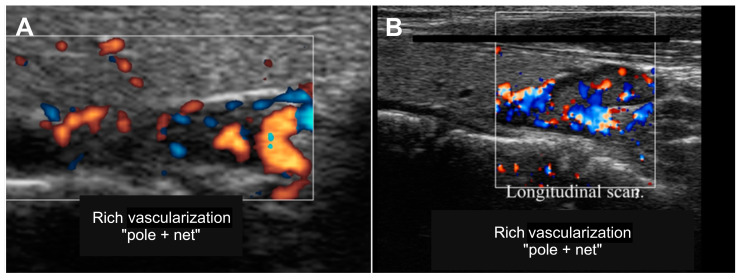
Ecocolor-Doppler US images of parathyroids (white rectangle), showing well-vascularized arterial pole (**A**) and arterial reticular pattern (**B**).

The high vascularization of the parathyroid glands helps differentiate them from lymph nodes and thyroid nodules. Indeed, the lymph nodes present vascularization with hilus located centrally or paracentrally, while the parathyroids show a peripheral arterial pole usually associated with a reticular parenchymal vascularization (Figure 2).

Neoplastic pathological lymph nodes may show radial vascularity in the peripheral part and disordered vascularity in the central part thus differentiating them from both normal and pathological parathyroid glands, with the possible exception of rare parathyroid carcinomas. Thyroid nodules often have poor vascularity, sometimes mainly arranged like a “basket” in the peripheral part, and sometimes internally distributed and even accentuated, but always in the absence of detectable vascular poles.

The utilization of a US contrast medium seems to be useful, albeit its adoption remains relatively limited in this clinical context.

US offers very high spatial resolution, allowing for a detailed evaluation of spatial relationships with other anatomical structures. It is highly available in medical settings and boasts a short examination time, typically around 20–30 min. Additionally, it is non-invasive and harmless, making it a safe option for patients with MEN1. US also provides the possibility to guide fine needle aspiration procedures (FNA) [14].

Despite its strengths, US has limitations. It is unable to study the mediastinum and to detect gland hyperfunction with certainty, lacking certain differential diagnosis capabilities in this regard, which could be especially relevant in patients with MEN1. Ultrasounds may also have difficulty detecting smaller or abnormally located parathyroid lesions (i.e., retrotracheal or in the subjugular region), especially in cases of hyperplasia where multiple glands are affected and may vary in size. Additionally, it may present difficulties in cases of atypical pattern of appearance, in the case of intrathyroidal glands, or in patients with “short neck” or bulky goiters.

**Figure 3:**
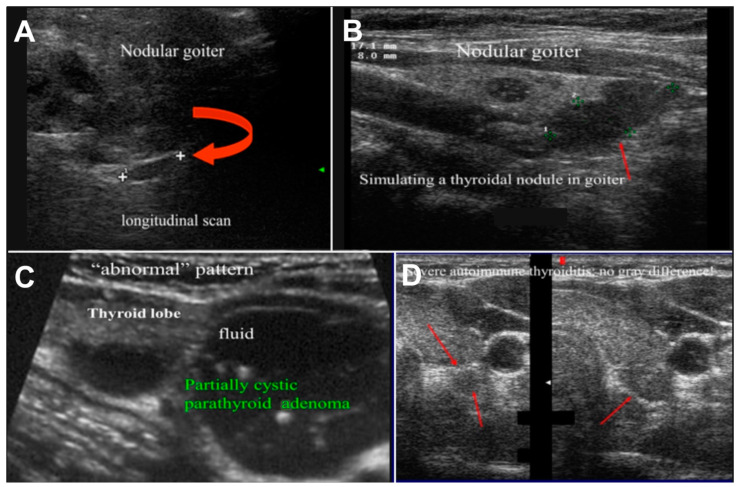
Challenging cases in US imaging. (**A**) Small parathyroid adenoma (red arrow) in patient with nodular goiter; (**B**) parathyroid adenoma (red arrow) simulating thyroidal nodule in patient with nodular goiter; (**C**) partially cystic parathyroid adenoma in patient with colloid-cystic goiter; and (**D**) parathyroid adenoma in patient with chronic thyroiditis: note isoecogeneity between thyroid and parathyroid gland.

US examination is also more difficult in situations that are particularly common in patients with MEN1, such as thyroid nodules, previous thyroidectomy or non-radical parathyroidectomy, or the presence of other neck masses (i.e., lymphadenopathies, neurinomas) (Figure 3).

Finally, a tumor may be the presence of thyroiditis (especially in chronic disease) or thyroid cancer, another relevant situation in patients with MEN1 [15]. In this case, the reduction in contrast between thyroid and parathyroid tissue and loss of thyroidal edges may significantly affect the detection rate. Moreover, in thyroiditis or thyroid cancers, perithyroidal lymph nodes may be enlarged and may show irregular shape thus posing another differential challenge. Consequently, the performance of US in detecting the parathyroid glands reported in the literature vary widely. For example, a 2012 meta-analysis of 19 studies evaluating US in patients with primary hyperparathyroidism revealed a pooled sensitivity of 76% and PPV of 93% [13], while other smaller studies reported sensitivities ranging from 44% to 97% [16,17,18].

Furthermore, in patients with recurrent hyperparathyroidism after parathyroidectomy, the sensitivity of US before reintervention is even lower, ranging from 46 to 69% [19,20].

Despite its limits, US associated with ecocolor-Doppler is a powerful tool in parathyroid detection and localization and it remains a fundamental technique in this clinical field.

## 3. Computed Tomography

The advent of multiphase parathyroid CT scanning, referred to as 4D-CT (four-dimensional computed tomography), has brought new hope for improved preoperative localization in pHPT cases. Its ability to quickly provide accurate multiplanar and dynamic imaging facilitates the precise identification of enlarged parathyroid glands, especially in the presence of ectopic or supernumerary glands. Moreover, in patients with MEN1 syndrome who underwent an initial parathyroidectomy but developed recurring hyperparathyroidism (up to 35% of cases) [21,22], the first intervention may have disrupted anatomical planes and resulted in variable amounts of scar tissue. In such settings, CT images provide superior anatomic information to identify the recurrent enlarged parathyroid and its relationship with surrounding neck and chest structures. CT images can also aid the surgeon in protecting the recurrent laryngeal nerve during intervention. CT can display anatomic arterial variants (such as an aberrant right subclavian artery or a right aortic arch with an aberrant left subclavian artery) linked to the presence of a non-recurrent laryngeal nerve, which is at increased operative risk of injury, especially if the surgeon is not preoperatively alerted.

Moreover, 4D-CT is performed by acquiring contiguous axial images of 1 mm thickness from the mandibular angles to the carina, including potential sites of ectopic glands. A three-phase protocol is generally used as recommended by the American College of Radiology appropriateness criteria [22], consisting of non-contrast material-enhanced, arterial (beginning 25–30 s after the start of contrast material injection), and late venous (beginning 80–90 s after the start of contrast material injection) phases. Iodinated contrast media is injected with a flux rate of 4 mL/s, and the bolus tracking technique improves the accuracy of the timing of the arterial phase, especially in older patients with decreased cardiac output. Table 1 shows the full acquisition parameters used at our institution.

**Figure 4:**
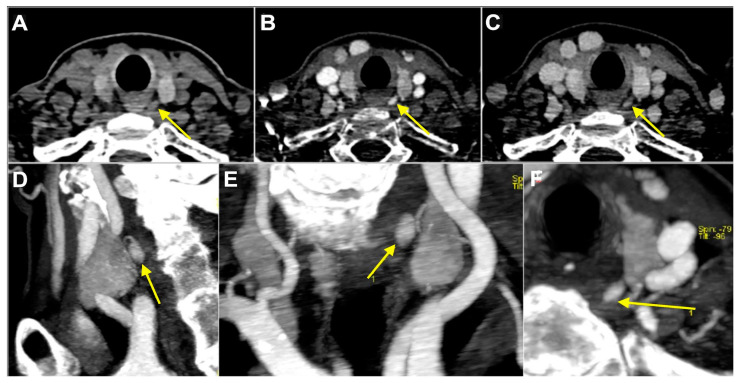
Enlarged left superior parathyroid in 53-year-old woman with primary hyperparathyroidism. Yellow arrow indicates 9 mm soft tissue nodule located posteriorly to third medium of left thyroid lobe, hypodense to thyroid parenchyma on non-contrast-enhanced phase (**A**), hyper enhancing on arterial phase (**B**) with subsequent wash-out on delayed phase (**C**). Reformatted sagittal (**D**), coronal (**E**), and axial (**F**) maximum intensity projection images obtained by arterial phase scan demonstrate upper enlarged polar vessel.

Multiplanar and maximum intensity projection (MIP) images are then evaluated. The classic appearance of an enlarged parathyroid gland on 4D-CT scans is an ovoid soft tissue lesion with the longest diameter greater than 6 mm, hypodense to the thyroid tissue on the non-contrast phase with greater contrast enhancement on the arterial phase and wash-out on the delayed phase (Figure 4).

An enlarged polar vessel entering the lesion is usually present and can be demonstrated well on MIP images. Cystic changes can be seen on larger lesions (>1 cm), and if present, they increase the confidence of lesion identification.

Sometimes, the enlarged parathyroid may present an atypical appearance, showing similar or lower enhancement than the thyroid on the arterial phase, and, occasionally, neither hyper enhance on the arterial phase nor wash-out on the delayed phase. In such cases, distinguishing a parathyroid from a lymph node or thyroid tissue can be challenging, and all three phases must be accurately examined [23].

Moreover, the possible intrathyroid location of a parathyroid gland can pose a diagnostic dilemma, especially in patients with multinodular thyroid struma or chronic hypothyroidism (Hashimoto disease) and might require additional imaging or anticipated surgical four-gland exploration.

A recent meta-analysis evaluating the diagnostic performances of 4D-CT for the preoperative localization of enlarged parathyroid gland shows per-lesion pooled sensitivity and specificity, respectively, of 81% (95%CI: 70–90%) and 85% (95%CI: 50–97%), and a positive predictive value (PPV) of 91% (95%CI: 82–98%) [7].

While several studies have demonstrated the superiority of 4D-CT over traditional imaging modalities in localizing parathyroid adenomas and multiglandular disease [8], some limitations exist.

Radiation exposure remains a concern, particularly in MEN1 and young patients or those requiring repeated imaging. The prevalence of malignant neoplasms, particularly thyroid and breast cancer, is higher in patients with primary hyperparathiroidism and MEN 1 (up to 7%) than the general population, so radiation exposure should be maintained as low as possible in order to minimize such risk [24]. The use of automatic tube current modulation leads to variability in CT radiation dose output; however, the effective dose usually ranges between 19 and 28 mSv [25].

Efforts to mitigate radiation exposure were obtained by using dual-energy 4D-CT, which allows for the generation of virtual non-contrast images and eliminates the need to obtain unenhanced acquisition. Using this technique, some studies [26,27,28] found similar sensitivities for lesion identification compared to conventional 4D-CT scans, with significant dose reduction.

Other limitations of using 4D-CT are related to the intravenous administration of iodinated contrast material, which carries the potential risk of contrast-induced nephropathy and allergic reactions.

Despite current limitations, ongoing advancements in imaging technology hold promise for further enhancing the utility of 4D-CT in pHPT management. Continued research efforts should focus on optimizing radiation dose protocols, improving image resolution, and exploring integration with emerging techniques such as artificial intelligence for automated gland detection.

## 4. Magnetic Resonance Imaging

MRI can potentially complement or even replace traditional imaging modalities for parathyroid localization in patients with pHPT. MR imaging generally allows for more precise tissue characterization via the acquisition of different imaging parameters (such as T1, T2, diffusion, and perfusion) that reflect different tissue characteristics [9]. Furthermore, MRI does not involve ionizing radiation, making it a safer option, particularly for vulnerable patient populations such as patients with MEN1. However, some challenges persist and limit the use of MR as the first-line imaging technique.

MR imaging for parathyroid localization is demanding both for physicians and patients. Image acquisitions can last several minutes, and the quality is often affected by artifacts related to both voluntary and involuntary patient movement (such as breathing, swallowing, and heartbeats reflecting along the chest and neck arteries). Moreover, imaging the thoracic inlet and the mediastinum can be challenging because of aliasing and interface artifacts, and obtaining a uniform adipose tissue suppression (in the fat-suppressed sequences) can be difficult due to the change in body morphology between the neck and the shoulders. Furthermore, the spatial resolution of MRI is not as high as that of 4D-CT, so outlining a parathyroid tumor with a few millimeters of thickness can be complicated.

**Figure 5:**
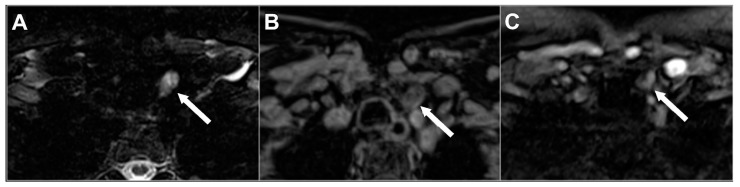
Left inferior parathyroid adenoma in 62-year-old woman with pHPT. Fat-suppressed axial T2w (**A**), pre-contrast axial T1w (**B**), and arterial frame on dynamic contrast-enhanced imaging (**C**) acquired on 1.5 T scanner. Parathyroid adenoma (white arrow) appears hyperintense on T2w and hypointense on pre-contrast T1w with heterogenous signal intensity and arterial enhancement due to internal cystic changes. Overall image quality affected by aliasing and motion artifacts.

The main characteristic of the hyperplastic and adenomatous parathyroid glands is the high signal intensity on T2-weighted images, usually well visible on fat-suppressed sequences. The appearance could be homogenous or patchy [9], especially when the lesion is larger than 1–1.5 cm, reflecting the presence of hemorrhagic foci, cholesterol clefts, and fibrosis (Figure 5).

However, a high T2-weighted signal intensity could also be present in exophytic thyroid nodules and lymph nodes, so some studies suggest including in the exam protocol both diffusion-weighted imaging and dynamic contrast-enhanced imaging (DCE) [10,29]. In particular, DCE imaging allows for the evaluation of the time–signal intensity perfusion curve and quantitative parameters, including time-to-peak enhancement, wash-in, and wash-out, that seem capable of distinguishing parathyroid adenoma from thyroid tissue or lymph nodes with an accuracy of 96% [29]. Moreover, DCE imaging demonstrated good sensibility not only in identifying single gland disease (92%) but also multigland disease (74%) [30].

Even though these newer MR imaging techniques show good potential, they require MR hardware and software with high technical demand and there is still limited broad availability across imaging centers. Moreover, further study with a larger sample size is necessary before MR imaging can regularly replace the other imaging modalities.

## 5. Nuclear Medicine Imaging

Radionuclide parathyroid imaging is a valuable tool in preoperative planning and is capable of detecting hyperfunctioning parathyroid tumors in typical and ectopic locations. The main nuclear medicine imaging techniques used include radiotracers for both scintigraphy/SPECT and PET imaging.

### 5.1. Scintigraphy and SPECT Imaging

[^99m^Tc]Tc-hexakis-(2-methoxy-2-isobutyl isonitrile ([^99m^Tc]-MIBI) is the oldest and commonly used radiopharmaceutical for parathyroid scintigraphy, accumulating in parathyroid tumors due to their increased mitochondria count [31]. [^99m^Tc]-MIBI exhibits higher uptake per gram in parathyroid tissue compared to thyroid tissue. Moreover, it typically washes out more rapidly from the thyroid than from parathyroid tumors, making it suitable for a dual-phase method with a single tracer administration [31]. The administered radioactivity varies between 400 and 900 MBq, based on factors like patient body mass and whether SPECT is conducted (Table 1) [11]. When CT is combined with SPECT, it is important to use the lowest CT dose compatible with the purpose.

[^99m^Tc]-MIBI SPECT/CT with dual-phase and subtraction technique is the standard first-line method for detecting parathyroid tumors, showing a high detection rate (88%) in patients with pHPT [32,33].

Scintigraphy with [^99m^Tc]-MIBI involves imaging at two time points (at 10–15 and at 90–150 min) after administration. SPECT/CT is considered superior to planar imaging, and at least one SPECT/CT study covering the area between the skull base and the heart base is recommended [34].

Another less used radiopharmaceutical, [^99m^Tc]-tetrofosmin, behaves similarly to [^99m^Tc]-MIBI, accumulating in the mitochondria of parathyroid lesions [35]. Since this tracer has no differential wash-out between parathyroid and thyroid tissues, it is suitable only for a dual-tracer subtraction method thus differentiating thyroid tissue from parathyroid tissue using administration of another thyroid-specific radiopharmaceutical [11].

### 5.2. PET Imaging

In most recent years, several different PET radiopharmaceuticals were also proposed in patients with pHPT, mostly because of the higher spatial resolution of PET/CT imaging thus improving the identification of even the tiniest pathological glands.

The most widely used PET tracers are choline analogs, especially (N-[(^18^F)fluoromethyl]-2-hydroxy-N,N-dimethylethanaminium ([^18^F]fluorocholine or [^18^F]FCH).

Choline serves as a marker for cellular proliferation, since it is a precursor of phosphatidylcholine, a key component of cell membranes. The stimulation of phospholipid-dependent choline kinase leads to the heightened uptake of [^18^F]FCH and is associated with parathyroid hormone secretion in pHPT [12,36].

Administered activity typically ranges from 100 to 300 MBq or 1.5–3.2 MBq/kg of body mass (Table 1). The optimal imaging time is 1 h post-administration of the radiopharmaceutical, eventually preceded by imaging obtained 5 min after injection, as some lesions may exhibit uptake only in the early phase [36]. Another option includes acquisition 20 min post-injection, with delayed images if the initial results are negative [37]. The field of view can be limited from the nose to the chest, down to the base of the heart, to minimize radiation exposure from CT. However, it can be readily extended to encompass the entire body based on the patient’s medical history (e.g., neoplasia, skeletal disease, infection) or any incidental findings.

**Figure 6:**
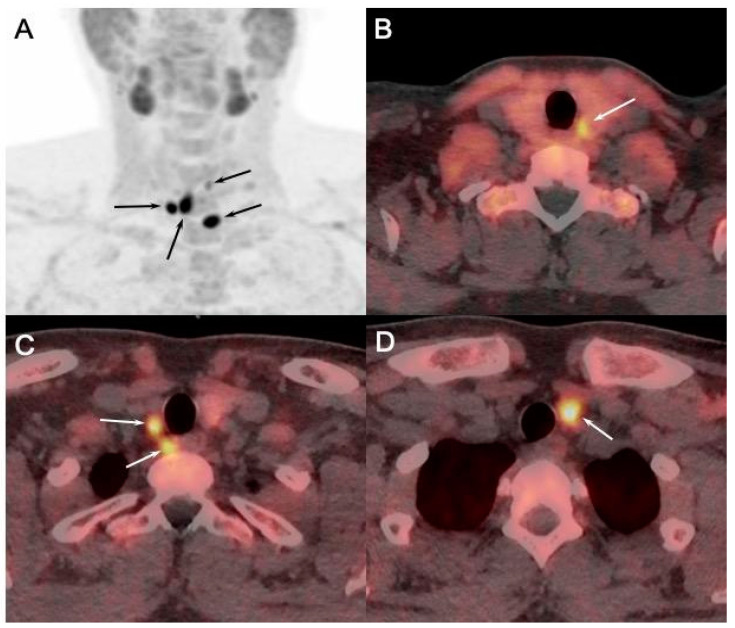
[^18^F]FCH PET/CT ((**A**): anterior MIP; (**B**–**D**): axial fusion images) performed in 23-y-old MEN1 patient with asymptomatic primary hyperparathyroidism (PTH: 389 ng/L; calcemia: 2.71 mmol/L) showing pathologic [^18^F]FCH uptake in both superior and inferior parathyroids (arrows). Pathology after surgical excision confirmed presence of parathyroid adenomas.

PET/CT utilizing ^18^F-labeled choline analogs demonstrates excellent results, with detection rates exceeding 90%, and reported sensitivity of 95% and PPV of 97% on a per-patient analysis, and 92% and 92% in a per-lesion analysis, respectively [36]. A recent meta-analysis reported the superiority of [^18^F]FCH PET/CT compared to conventional imaging methods, with a sensitivity of 92% for [^18^F]FCH PET/CT and 65% for a combination of conventional methods in the setting of sporadic sHPT (Figure 6) [12].

Additional benefits of PET/CT using [^18^F]FCH compared to [^99m^Tc]-MIBI scintigraphy include reduced radiation exposure [38], enhanced resolution, and shorter acquisition duration. Consequently, it is regarded as an alternative primary imaging modality [39,40,41,42,43].

Other PET tracers are available for parathyroid imaging, although they are not used frequently in clinical practice. [^11^C]2-hydroxy-N,N,N-trimethylethanamium ([^11^C]CH), another choline analog, exhibits strong uptake in parathyroid cells. Advantages include rapid background activity clearance, shorter acquisition time (30 min), and low radiation exposure [11,44]. L-[methyl-^11^C]methionine ([^11^C]MET) is an aminoacidic analog trapped in parathyroid tumors and is involved in the synthesis of the PTH precursor. Both administered activities, reported acquisition times and sensitivity, are highly variable [44].

The outstanding accuracy of [^18^F]FCH PET/CT in identifying parathyroid tumors in sporadic pHPT, surpassing that of [^99m^Tc]-MIBI scintigraphy and SPECT/CT, was verified by numerous research groups globally [45], as well as through meta-analyses [12] and recent EANM guidelines [11]. However, in the setting of MEN1, studies examining the efficacy of ^18^F]FCH PET/CT compared to other imaging techniques like parathyroid US and [^99m^Tc]-MIBI scintigraphy in pinpointing parathyroid tumors are relatively scarce and challenging due to limited sample sizes and patient heterogeneity, often spanning an extensive timeframe.

**Figure 7:**
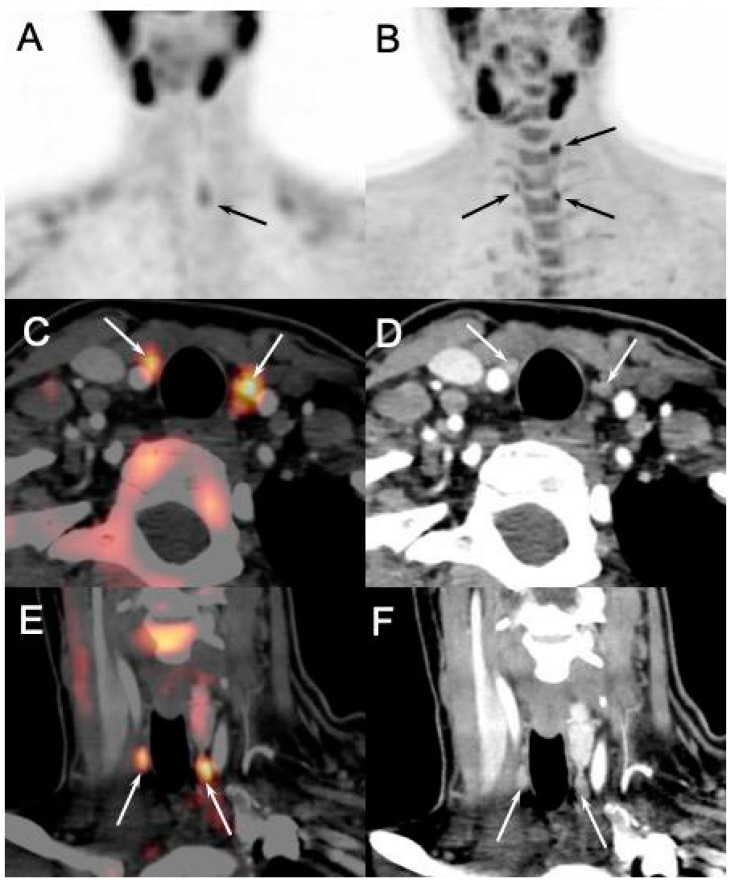
Discordant results of [^99m^Tc]sestamibi parathyroid scintigraphy ((**A**): anterior MIP) and [^18^F]FCH PET/4D-CT ((**B**): anterior MIP; (**C**,**E**): axial and coronal fusion images; (**D**,**F**): axial and coronal 4D-CT) performed in 28-y-old MEN1 patient with recurrent primary hyperparathyroidism (PTH: 133 ng/L; calcemia: 2.70 mmol/L). [^18^F]FCH PET/CT confirmed scintigraphy findings (pathological left inferior gland) and detected two more hyperfunctioning parathyroids tumors (arrows, inferior right and superior left) afterwards confirmed by pathology.

In this setting, recent studies showed not only a high global patient-based positivity rate of imaging (91% for parathyroid US and 96% for [^99m^Tc]-MIBI scintigraphy and SPECT/CT and [^18^F]FCH PET/CT) [46], but also an added value of the latter imaging modality. Indeed, it was demonstrated that preoperative [^18^F]FCH PET/CT provides additional surgically relevant insights compared to the other imaging modalities, revealing additional pathologic glands (Figure 7), the majority of which were not detected by parathyroid US.

**Figure 8:**
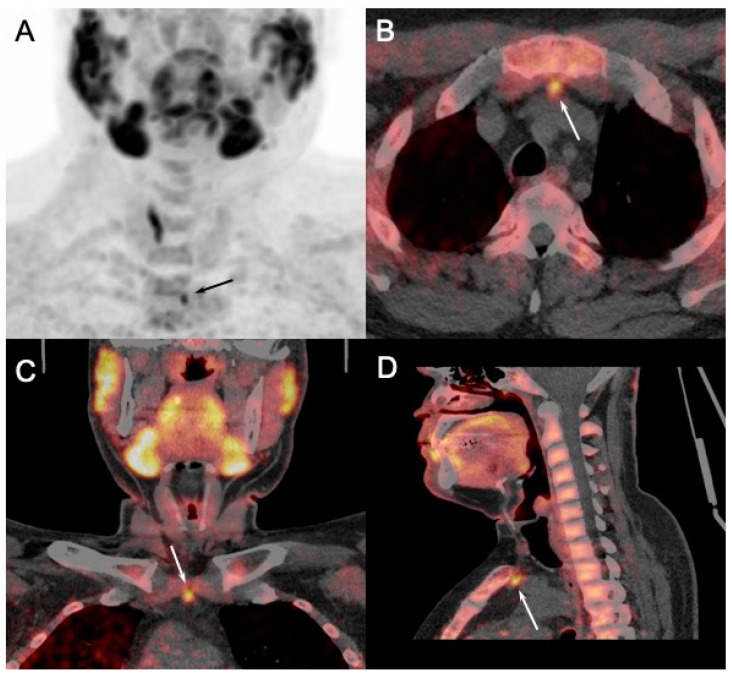
[^18^F]FCH PET/CT ((**A**): anterior MIP; (**B**–**D**): axial fusion images) performed in 40-y-old MEN1 patient with recurrent primary hyperparathyroidism (PTH: 290 ng/L; calcemia: 2.66 mmol/L) and previous history of left thyroidectomy, superior left, and inferior bilateral parathyroidectomy. Cervical US failed to detect pathological glands. PET/CT showed pathologic [^18^F]FCH uptake in 8mm retrosternal soft tissue nodule, suggesting ectopic parathyroid tumor afterwards confirmed by pathology after surgical excision.

Moreover, in a preoperative setting, [^18^F]FCH PET/CT seems more accurate and useful than [^99m^Tc]-MIBI scan in pHPT patients with positive scintigraphic results [47]. In cases of multiglandular hyperplasia, a frequent condition in patients with MEN1, the four parathyroid glands never exhibit the same intensity of uptake on [^18^F]FCH PET. Consequently, the supplementary information offered by [^18^F]FCH PET/CT could facilitate better decision-making regarding the conservation or cytoreduction of parathyroid glands based on their level of hyperfunction assessed by the SUVmax. It has also enhanced surgical exploration by targeting abnormal and ectopic locations [48] (Figure 8).

In operated patients with MEN1, the sensitivity of [^18^F]FCH PET/CT stands between 76% and 100%, surpassing that of [^99m^Tc]-MIBI scintigraphy and SPECT/CT, yet falling short of the reported sensitivity for sporadic pHPT (92%) in some studies [47,48]. This discrepancy could be attributed to a higher prevalence of hyperplastic parathyroid glands (69%) in MEN1 subjects, which are associated with a lower reported sensitivity [12].

Another clinical challenge in patients with MEN1 is the heightened recurrence rate following parathyroidectomy, compared to sporadic pHPT. The persistence of pHPT over many years often necessitates repeated resections in the lifespan of patients with MEN1, presenting a delicate balance between persistent pHPT and lifelong hypoparathyroidism in the event of excessive resection. In this particularly demanding scenario, [^18^F]FCH PET/CT displayed an even higher detection rate compared to the same examination conducted on patients prior to parathyroidectomy. Specifically, among patients with MEN1 with surgical history, [^18^F]FCH PET/CT identified suspected foci of pathological parathyroid tissue in almost all patients [49]. Furthermore, among patients who underwent re-operation, [^18^F]FCH PET/CT successfully detected all resected parathyroid tumors (100%), additionally guiding surgical procedures, enabling minimally invasive unilateral exploration, bilateral exploration, or completion via thoracoscopy. Importantly, none of the patients who underwent re-operation experienced persistent pHPT. Such an outstanding outcome might stem from the observation that, in cases of recurrent post-surgery pHPT, parathyroid adenomas were more common histologically than hyperplasia [48,49].

**Figure 9:**
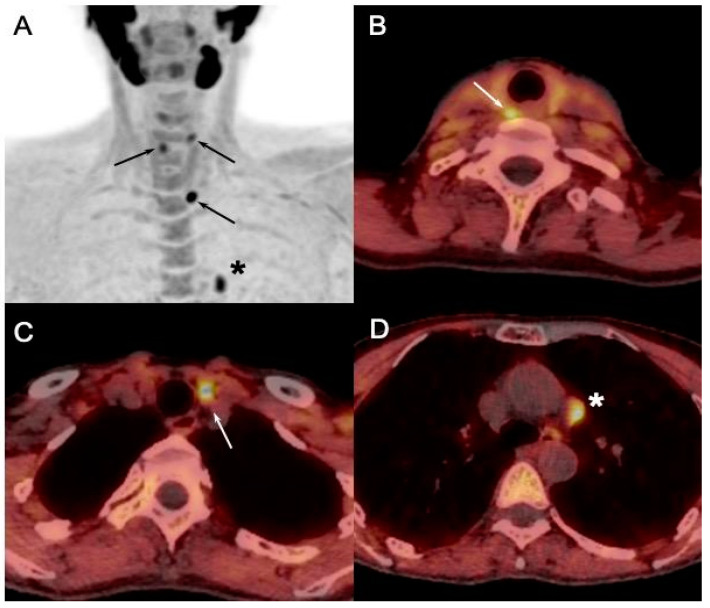
Initial staging in MEN1 patient with primary hyperparathyroidism (PTH: 157 ng/L; calcemia: 2.78 mmol/L) and probable left inferior parathyroid adenoma at cervical US. [^18^F]FCH PET/CT ((**A**): anterior MIP; (**B**–**D**): axial fusion images) confirmed US findings and detected two additional pathological parathyroids (arrows, superior right and left). In addition, hypermetabolic soft tissue nodule (*) of about 10mm was detected in left mediastinum, which suggested pathologic ectopic parathyroid, but turned out to be thymoma (B2) after surgery.

False positive findings can occur during total-body [^18^F]FCH PET/CT scans, especially in regions of parathyroid ectopy, where the majority may be thymic tumors (Figure 9). Although these findings may be deemed false positives in imaging, they remain noteworthy because diagnosing such tumors in patients with MEN1 carries a poor prognosis and may alter the intended surgical approach [48,49].

Additional neoplastic conditions that may manifest as positive findings for [^18^F]FCH PET/CT in patients with MEN1 include pituitary neuroendocrine neoplasms and adenomas, brown tumors [50], and breast cancer [51]. Recognizing these conditions is especially crucial for comprehensively managing MEN1-associated morbidity.

Among potential pitfalls of PET/CT using ^18^F-labeled choline analogs is the uptake by inflammatory lymph nodes and thyroid nodules, which could lead to false positive results [52].

The combined application of [^18^F]FCH PET/CT and 4D-CT in an integrated fashion has attracted considerable attention as a comprehensive approach to preoperative localization in pHPT [53,54]. This integrated imaging approach seeks to capitalize on the distinct advantages of each technique, potentially enhancing localization precision and streamlining surgical planning [55]. While its effect on sensitivity remains debatable, the concurrent utilization of [^18^F]FCH PET and 4D-CT in a unified examination holds promise in assisting with differential diagnosis, potentially lowering the incidence of false positive PET findings [56]. Recent data highlighted the interest of a preoperative approach for primary hyperparathyroidism (pHPT) by prioritizing [^18^F]FCH PET/CT as the initial modality, followed by sequential 4D-CT during the same session if PET/CT findings are negative or inconclusive. This tailored strategy may improve the localization of pathological parathyroid glands in complex cases. In instances where PET/CT results are clearly positive, the added value of 4D-CT is minimal. Restricting the use of 4D-CT to cases with negative or inconclusive PET/CT findings preserves accuracy while minimizing radiation exposure and costs. However, further prospective studies with a larger cohort of negative or inconclusive cases are essential to fully evaluate the clinical advantages of this approach over standard algorithms [57].

Notably, hyperfunctioning parathyroid tumors could also be incidentally discovered in patients with MEN1 undergoing somatostatin receptor PET/CT with [^68^Ga]-DOTA-peptides, a PET imaging technique employed to localize different types of endocrine tumors. However, despite the potential for somatostatin receptor PET to detect parathyroid adenomas, its lesion-based sensitivity for this purpose is low (less than 30%). Therefore, while the identification of foci consistent with abnormal parathyroids on [^68^Ga]-DOTA-peptides PET/CT should trigger the biochemical evaluation for pHPT, it cannot be regarded as a preoperative localization method [58].

Lastly, a drawback of all nuclear medicine imaging and also of CT imaging is the exposure to ionizing radiation. This poses a particular concern in patients with MEN1 because menin plays a role in DNA repair, cell cycle control, and transcriptional regulation. Hence, there exists a theoretical risk, at minimum, that repeated exposure to ionizing radiation in patients with a hereditary defect in a tumor suppressor gene may heighten the oncogenic risk [59].

**Figure 10:**
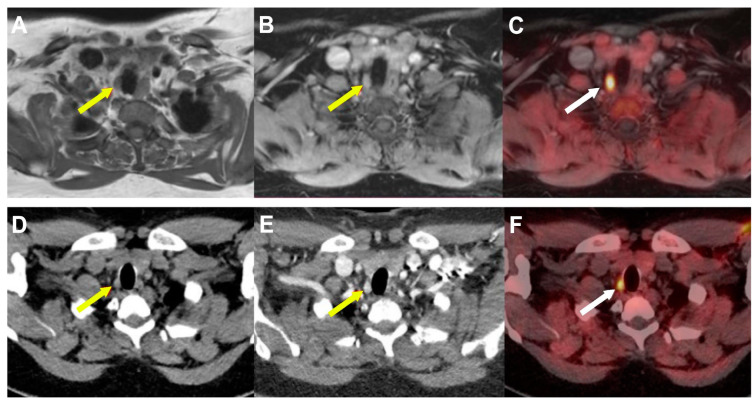
Concordant results of [^18^F]FCH PET/4D-MRI and [^18^F]FCH PET/4D-CT (axial slices) in patient with primary hyperparathyroidism showing right inferior parathyroid tumor (arrows) with high [^18^F]FCH uptake, and early and intense contrast media enhancement ((**A**): T2-weighted MRI; (**B**): arterial phase T1-weighted MRI; (**C**): PET/MRI fusion images; (**D**): no contrast media enhanced CT; (**E**): contrast media enhanced CT (arterial phase); (**F**): PET/CT fusion images). Parathyroid adenoma confirmed after surgery.

Notably, MRI procedures involve no patient exposure to radiation. Additionally, MRI offers excellent soft tissue contrast and has the potential to offer superior differentiation compared to CT between parathyroid glands, muscle tissue, and other soft tissues such as lymphatic structures (Figure 10). Consequently, [^18^F]FCH PET/MR could represent a good compromise and a progressive advancement in evaluating patients with MEN1 and pHPT [60,61], although it remains premature to delineate its advantages compared to conventional imaging techniques.

**Table 1:** Protocols for different imaging techniques and corresponding effective dose.

Imaging Technique	Protocol	Effective Dose (mSv)
Neck ultrasound	Linear multi-frequency probe (3–15 MHz) equipped with ecococolor-Doppler (frequency range 500–1500 MHz)	0
4D-CT [23]	Scan coverage: maxilla to carina Acquisition slice thickness: 1 mm Tube voltage: 120 kV Tube current (automatic modulation): 100–400 mA (min-max) Contrast medium administration: 80 mL (370 or 400 mg Iodine/mL) at 4 mL/s followed by 30 mL saline flush Three phases: non-contrast enhanced, arterial (25–30″ after the start of contrast injection or 10″ with bolus tracking in the aortic arch), and delayed (80–90″) 1 mm sagittal and coronal reformats and 3 mm maximum intensity projection reconstructions	19–28
MRI	Scan parameters differ by site, magnet, and vendor: image quality best on 3T scanner Dixon technique is best used for homogenous fat saturation Contrast medium administration: 0.1 mmol/kg of Gadobutrol at 4 mL/s followed by 30 mL saline flush Core sequences: 3 mm fast spin echo fat-suppressed T2-weighted images on axial and coronal planes 3 mm fast spin echo T1-weighted images on the axial plane High resolution (gradient echo) pre and post-contrast (arterial and delayed phases) fat-suppressed T1-weighted images (2 or 3 mm thickness on the axial plane) Or Volumetric contrast-enhanced dynamic imaging (gradient echo) acquired for 120 s with a temporal resolution of 6 to 10 s (12 to 20 temporal frames) Added sequence: 4 mm axial diffusion-weighted imaging with b values of 50, 400, and 800 s/mm^2^	0
[^99m^Tc]-MIBI scintigraphy/SPECT [11]	Administered activity: 400–900 MBq Dual-phase acquisition at 10–15 and at 90–150 min after injection Scan coverage: neck and mediastinum	4.57
[^18^F]FCH PET/CT [11]	Administered activity: 100–300 MBq Acquisition: 1 h after injection (possibly preceded by imaging at 5 min) Scan coverage: maxilla to carina or total-body	4.00

Abbreviations: four-dimensional computed tomography (4D-CT), magnetic resonance imaging (MRI), [99mTc]Tc-hexakis-(2-methoxy-2-isobutyl isonitrile ([^99m^Tc]-MIBI), single photon computed tomography (SPECT), [^18^F]fluorocholine ([^18^F]FCH), positron emission tomography (PET).

## 6. Discussion

Given the advancements in minimally invasive surgical techniques, pinpointing the location of parathyroid glands is increasingly crucial, enabling meticulous surgical planning, with minimized surgical access and shorter operation times. Such accurate and precise localization is particularly of relevance for patients with MEN1. Moreover, due to the unique biological properties of parathyroid tissue in MEN1 individuals, a post-surgery reassessment of residual tissue location is often necessary if there is a recurrence of elevated parathyroid hormone levels. Hence, alongside the accuracy of each detection method, it is essential to consider both the exam’s repeatability and its safety (Table 2).

US stands out as the swiftest, safest, and effective initial imaging method for examining the neck and identifying abnormal parathyroid glands, especially when conducted by skilled radiologists. Nevertheless, research suggests that relying solely on US may not always suffice for detecting all pathological glands, a critical consideration in patients with MEN1 syndrome. [^18^F]FCH PET/CT offers a favorable benefit-to-risk ratio in detecting parathyroid tumors in MEN1 cases. It boasts the highest detection rate among current imaging modalities, particularly hyperplastic glands, with few drawbacks and a significant impact on patient management compared to other imaging modalities. Recent EANM guidelines now recommend it as a primary or secondary imaging modality. However, it could be considered as a first-line modality in patients with MEN1 due to their persistent pHPT, concerns about disease recurrence, and the critical need for precise gland localization during the initial operation for long-term cure. The main risk is radiation exposure, which is lower than that of [^99m^Tc]-MIBI and 4D-CT. The expected one to three [^18^F]FCH PET/CT scans over an MEN1 patient’s lifespan result in minimal radiation exposure compared to the mean effective dose of 121 mSv observed in a cohort of patients with MEN1 at a specialized institution between 2007 and 2015.

## 7. Perspectives

The choice of initial imaging for patients with MEN1 remains contentious even in centers with considerable expertise and caseloads. The diagnostic strategy for these patients should prioritize a personalized approach, aiming to identify all abnormal parathyroid glands, not solely adenomas. Our suggestion for an initial localization approach in patients with MEN1 involves a combination of US and [^18^F]FCH PET/CT. If this initial imaging yields inconclusive or conflicting results, 4D-CT or MRI can serve as valuable problem-solving tools.

## Figures and Tables

**Table 2:** Comparison of various imaging modalities used in primary hyperparathyroidism.

Imaging Modality	Advantages	Disadvantages
Neck Ultrasound [5,6,13,14,15,16,17,18,19,20]	- Non-invasive - Widely available - No radiation exposure - Cost-effective	- Operator-dependent - Limited in detecting ectopic glands - Lower sensitivity in patients with prior neck surgery
4D-CT [7,8,23,24,25,26,27]	- High spatial resolution - Excellent anatomical detail - Superior in detecting ectopic glands - Able to identify HPTs close to large goiter or with relevant cystic component - Widespread availability	- Relevant radiation burden - Requires sufficient renal function and cannot be performed in subjects allergic to contrast media - Limited effectiveness in multifocal disease
MRI [9,10,29,30]	- No radiation exposure - Excellent soft tissue contrast - Useful in patients with contraindications to CT contrast - Good for detecting ectopic glands	- Higher cost compared to ultrasound - Longer imaging time - May require gadolinium contrast, with potential for allergic reaction or nephrogenic systemic fibrosis in patients with renal impairment - Limited availability and expertise in some centers
[^99m^Tc]-MIBI scintigraphy/SPECT [11,32,33,34]	- Good sensitivity for detecting hyperactive parathyroid glands - Functional imaging with dual-phase technique - Can detect ectopic glands - Widely available	- Lower spatial resolution compared to CT - May miss smaller or less active adenomas - Requires prolonged imaging time - Radiation exposure
[^18^F]FCH PET/CT [11,12,36,37,38,39,40,41,42,43,44,48,49,50,51,52,53]	- High sensitivity and detection rates - Excellent for detecting small or ectopic parathyroid adenomas, or multifocal disease - Provides both metabolic and anatomical information - Faster imaging time compared to SPECT - Low radiation dose to patient - Able to identify further relevant findings in whole body	- Limited availability - High cost - Radiation exposure - Inferior anatomical definition - Less sensitive in HPTs with cystic component

Abbreviations: four-dimensional computed tomography (4D-CT), hyperparathyroidism (HPT), magnetic resonance imaging (MRI), [99mTc]Tc-hexakis-(2-methoxy-2-isobutyl isonitrile ([^99m^Tc]-MIBI), single photon computed tomography (SPECT), [^18^F]fluorocholine ([^18^F]FCH), positron emission tomography (PET).

## Data Availability

Review studies do not require registration.
